# Determining Factors and Zootechnical Output of Biosecurity Practices in Fish Farms in the Wouri Division, Cameroon

**DOI:** 10.1155/2023/2504280

**Published:** 2023-03-30

**Authors:** Fonkwa Georges, Makombu Judith Georgette, Kamdem Alex Henri, Kametieu Djamou Franck, Nack Jacques, Awah-Ndukum Julius, Tomedi Eyango Minette, Tchoumboue Joseph

**Affiliations:** ^1^Laboratory of Aquaculture and Demography of Aquatic Resources, Department of Aquaculture, Institute of Fisheries and Aquatic Sciences, University of Douala, P.O. Box 7236, Douala, Cameroon; ^2^Applied Hydrobiology and Ichthyology Research Unit, Department of Animal Science, Faculty of Agronomy and Agricultural Science, University of Dschang, P.O. Box 222, Dschang, Cameroon; ^3^Department of Fisheries and Aquatic Resources Management, Faculty of Agriculture and Veterinary Medicine, University of Buea, Buea, Cameroon; ^4^College of Technology, University of Bamenda, Bambili, Cameroon

## Abstract

Biosecurity practice limits the occurrence of diseases and economic losses in fish farms. The objective of this study was to characterize fish farming in the Administrative Division of Wouri, Cameroon (3°97′04″–3°58′13″N; 9°76′78″–9°46′4.3″E) and assess the biosecurity practices. A cross-sectional biosecurity audit was then conducted in 33 fish farms from March to May 2022. The “snow ball” technique, on-farm observations, and face-to-face interviews of farm managers using a semistructured questionnaire were used for data collection. The results showed that most of the fish farmers were between 18 and 40 years of age (63.64%) and not trained in fish farming (60.61%). The lack of finance (57.57%) was the main constraint to the biosecurity practice. The high fish mortality rate (>15%) was recorded in 66% of the farms. Overall, the compliance rate (CR = 40.52 ± 14.70%) and adoption rate (AR = 40.40 ± 30.10%) of biosecurity measures were intermediate. No type *C* farm or at the minor risk level of contamination was recorded. Farmers of 18 to 40 years of age (45.24 ± 14.75%) who attended higher school (43.83 ± 14.44%) and received training in fish farming (47.44 ± 14.39%) recorded a significant higher CR. The CR and AR were significantly higher for the isolation component (CR = 60.17 ± 19.81%; AR = 60.17 ± 25.68%) followed by traffic control (CR = 53.53 ± 25.87%; AR = 53.53% ± 34.86) and sanitation (CR = 27.70 ± 19.70%; AR = 29.84 ± 26.00%). A strong (*R*^2^ = 0.725), positive, and significant (*p*=0.019) linear relationship was found between the level of education of fish farmers and the biosecurity compliance rate while the health status of fish was weakly (*R*^2^ = 0.207), positively, and significantly (*p*=0.017) influenced by the compliance rate. Fish farming is an income-generating activity that still requires socioeconomic, technical, and institutional efforts for optimal productivity. The Cameroonian government should emphasize on the education, training, and capacity building of farmers on biosecurity practices to minimise the introduction, establishment, and spread of diseases.

## 1. Introduction

Fish farming is the main solution to meet world fish demand. It secures food supply and poverty alleviation and as an income-generating activity, the sector employed 304,000 Africans in 2016 [1]. In Cameroon, the annual fish production of 335,000 tons is far short of estimated demand of 500,000 tons/year and has caused supplementary yearly importation of about 180,000 tons of fish. Institutional, economic, technical, and infrastructural constraints to the development of fish farming sector have been reported [2]. No emphasis is laid on fish diseases responsible for the fish mortalities and the decrease of farms productivity. Though the evaluation of the economic impact of diseases in fish farming has not yet been the primary concern of researchers in the developing countries [3], the economic loss caused by fish diseases have been reported. Hence, from 2010 to 2017, reduction in salmon production and export due to necrotizing hepatopancreatitis amounted to USD 12 billion in Thailand and over USD 26 million in Viet Nam in 2015 [4].

The failure of compliance with biosecurity measures or hygiene has been declared to be at the origin of the transmission and spread of diseases in fish farms [5]. Biosecurity as a strategic and integrated approach that encompasses policy and regulatory frameworks aimed at analyzing and managing risks relevant to human, animal, and plant life and health, including associated environmental risks. Aquaculture biosecurity includes control of the spread of aquatic plant and animal diseases and invasive pests and the production of products that are safe to eat [6]. Thus, a sustainable management of the risks related to diseases in fish farms requires a thorough knowledge of all the factors likely to influence the implementation of biosecurity standards. Biosecurity is an essential tool to reduce the risk of diseases entering in a farm and suitable biosecurity practices can prevent emerging health issues, reduce impacts of disease, and improve profitability. Once the disease occurs, the treatment becomes technically and financially more demanding, hence the need of preventing diseases instead of a curative response [7, 8]. Apart from the report of Ngueguim et al. [9] on the biosecurity practices on fish farms in the West Region of Cameroon, no information relevant to the level of biosecurity implementation to assist policymakers in fish industry is available although farms in Wouri Division are regularly subjected to diseases and massive mortality of fish, namely, Nile tilapia (*Oreochromis niloticus*), common carp (*Cyprinus carpio*), and catfish (*Clarias gariepinus*). This study was therefore designed to characterize the fish farming and biosecurity practices, to assess the effects of the socioeconomic characteristics of fish farmers on the implementation level of biosecurity measures, and their zootechnical consequences in the Wouri Division.

## 2. Materials and Methods

### 2.1. Study Area

**Figure 1:**
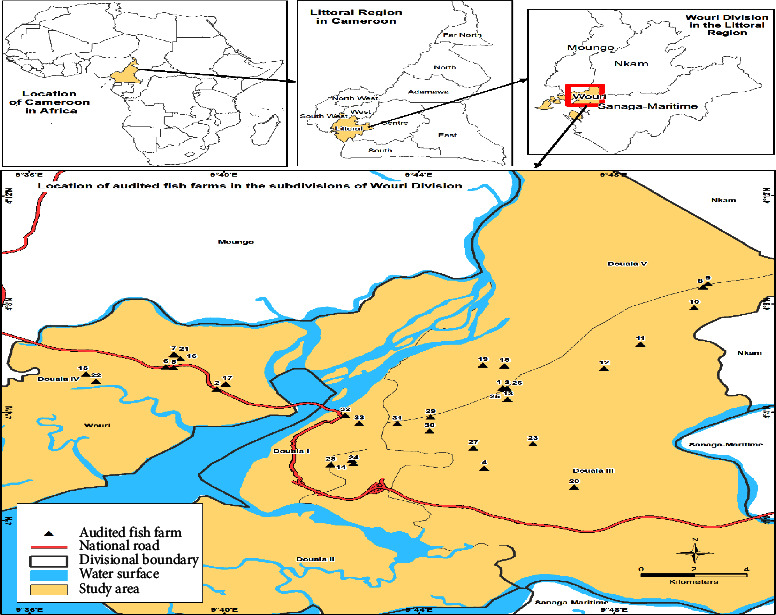
Location of audited fish farms in the Littoral Region of Cameroon.

The cross-sectional study was carried out from March to May 2022 on fish farms located in the administrative subdivisions (Douala I, Douala II, Douala III, Douala IV, and Douala V) of the Wouri Division (3°97′04″–3°58′13″N; 9°76′78″-9°46′4.3″E), the Littoral Region of Cameroon (Figure 1). The subdivisions were selected based on the importance of fish farming in these locations as advised by the Wouri Delegation of Livestock, Fishery and Animal Industries. The climate is of the equatorial type with a rainy season (March–November) and a dry season (December–February). The annual average temperature is 27.4°C and the rainfall of about 3619 mm [10].

### 2.2. Selection of Fish Farms

Since the official registry of fish farms was lacking, they were first located with the help of a local inhabitant. The next farm was identified using the “snow ball” technique [11] during which the manager of the previously audited farm was asked to indicate the neighboring farm and so on until the entire area was completely covered [12]. Eligibility criteria to participate in the study for fish farms taken into consideration were road accessibility, functional status, and availability of the farm manager [5]. A total of thirty-three (33) farms were audited and codified.

### 2.3. Questionnaire Design and the Biosecurity Scoring System

The data were collected by on-farm observations and face-to-face interviews of farm managers using a semistructured questionnaire divided into three parts. The first part was composed of the socio-economic characteristics of fish farmers (age, sex, marital status, number of years of exploitation, educational level, training in fish farming, place of training, main occupation, purpose of fish farming, mode of land acquisition, constraints, and cost related to the biosecurity practice). The second part entailed the zootechnical characteristics of the farm (the sanitary status of the fish, mortality rate, and productivity) while the third section was made up of the twenty-four (24) biosecurity measures grouped into components, namely, isolation, traffic control, and sanitation [13] modified from Arthur et al. [14] and adapted to the present study. The questionnaire was previously tested in a subsample of seven (7) farms in the study area to verify the relevance, clarity, redundancy, and consistency of the questions. Subsequent adjustments were made accordingly. The geographical coordinates of the farms were recorded using a GPS (the Global Positioning System).

The linear scoring or weighting (0–1) of the biosecurity measures was used. Thus, the values 1 and 0 were assigned, respectively, to the biosecurity measure implemented or not. The final score of a biosecurity measure was the sum of all the values recorded in the farms (0 or 1 per farm). Since a biosecurity component (isolation, traffic control, and sanitation) included several measures, the average score of a component was the ratio between the sum of the score of its measures and the number of biosecurity measures of the component. The maximum score of a given measure and farm was 33 and 24 points, respectively. The linear scoring system was empirically calculated as previously described [15–19]. In fact, the measures were weighted equally and any biosecurity measure estimated to be less efficient in the transmission and occurrence of a disease since fish may suffer from poor health due to lack of implementing biosecurity measures. The focus was on the importance of implementing biosecurity measures on the health of farmed fish and not the level of risk generated by each biosecurity measure as it is the case in disease transmission pathways. The weighed scoring systems in the disease transmission pathways should not have the same efficiency given that direct contact is likely more risky than indirect contact with less efficiency for transmitting pathogens.

### 2.4. Determination of the Compliance and Adoption Rates of Biosecurity Measures

The compliance rate (CR) and adoption rate (AR) of biosecurity measures were defined after Racicot and Vaillancourt [5].

(1) $$\begin{array}{c} AR=\frac{Number\;of\;farms\;applying\;a\;biosecurity\;measure\;\left(Total\;score\;of\;the\;measure\right)}{Total\;number\;of\;audi\;ted\;farms}X\;100\text{,}\\ CR=\frac{Number\;of\;measures\;applied\;by\;a\;farmer\;\left(Total\;score\;of\;the\;farm\right)}{Total\;of\;recommende\;d\;measures}X\;100\text{.} \end{array}$$

The ranking of the compliance rate (low, intermediate, and high) recommended by Racicot and Vaillancourt [5] was equally applied to the adoption rate and adapted to the present study to classify fish farms (Table 1).

### 2.5. Statistical Analysis

Data were stored in the Excel spreadsheet (Microsoft Office 2010, USA) and exported to R software for analysis. The socioeconomic and zootechnical characteristics and the compliance and adoption rates were subjected to descriptive statistics. The mean values of compliance and adoption rates were compared using the Kruskal–Wallis *K* test, the Mann–Whitney *U* test , and the analysis of variance (F). The multivariate linear regression model was used to assess the relationship between biosecurity scores and socioeconomic and zootechnical characteristics of fish farmers and farms, respectively. Biosecurity affinities (similarities) and interactions between fish farms were determined using the principal component analysis (PCA). The significance level (*p*) was set at 0.05.

## 3. Results

### 3.1. Socioeconomic and Demographic Characteristics of Fish Farmers in the Wouri Division

The distribution of farms frequencies according to the socioeconomic and demographic characteristics of fish farmers in the Wouri division (Table 2) shows that more than half of fish farmers (63.64%) were between 18 and 40 years of age. In addition, men (93.94%) were more involved in fish farming. Farmers were mostly married (54.54%) and nearly 75% of them completed higher education. Out of 39.39% of farmers who received training in fish farming, 53.85% came from the Institute of Fisheries and Aquatic Sciences (ISH), University of Douala, Cameroon. The main constraint related to the biosecurity practice was the high cost (57.57% of farms). Ignorance and neglect of the biosecurity practice accounted only for 15.15 and 9.09%, respectively, of fish farmers.

### 3.2. Zootechnical Characteristics of Fish Farms in the Wouri Division

The zootechnical characteristics of fish farms in the Wouri division as summarized in Table 3 outline that signs of fish diseases were observed in 42.42% of farms. Nearly, 66% of the audited farms showed a high fish mortality rate (>15%) while few (12.12%) recorded productivity higher than 300 kg/year/m^3^.

### 3.3. Distribution of the Compliance Rate per Subdivisions of the Wouri Division

The distribution of the compliance rate per subdivisions of the Wouri division is summarized in Table 4. The overall compliance rate (40.52 ± 14.70%) was intermediate indicating that farms were of type B or at the moderate risk level of contamination by pathogens. The compliance rate ranged from 36.11 ± 15.78% (Douala I) to 42.80 ± 18.08% (Douala II) with no significant influence (*F* = 0.167; *p*=0.953) of the farms' location.

### 3.4. Compliance Rate in Relation to Biosecurity Components

**Figure 2:**
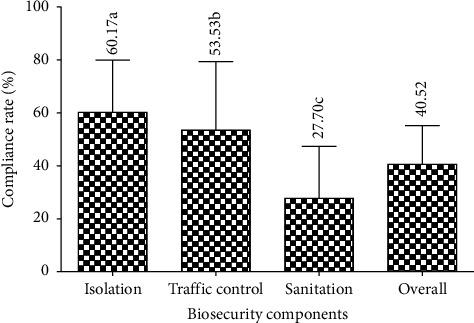
Compliance rate in relation to the biosecurity components.

The compliance rate in relation to biosecurity components (Figure 2) shows that the isolation (60.17 ± 19.81%) was significantly (*F* = 22.73; *p* < 0.0001) the most observed component followed by the traffic control (53.53 ± 25.87%) and sanitation (27.70 ± 19.70%).

### 3.5. Distribution of Farm Frequencies Relevant to the Compliance Rate of Biosecurity Components

**Figure 3:**
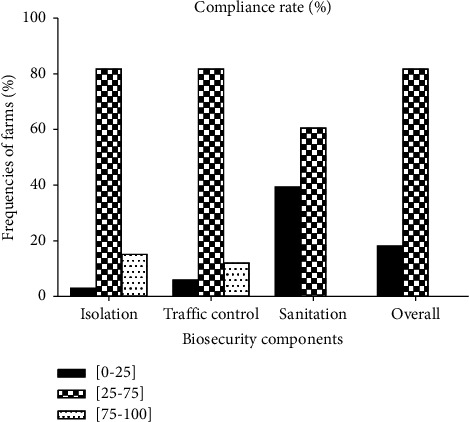
Distribution of farm frequencies relevant to the compliance rate of biosecurity components.

The distribution of farm frequencies relevant to the compliance rate of biosecurity components as illustrated in Figure 3 indicates that globally, 18.18% and 81.81% of audited fish farms recorded a low and intermediate compliance rate, respectively. No farm (0%) was of type C or at a minor risk level of contamination. Whatever the biosecurity component, the intermediate biosecurity practice was the most observed (75% of farms), while the poor biosecurity practice was the less implemented. The good biosecurity practice was only observed for the isolation and traffic control implemented by 15.15% and 12.12% of farms, respectively.

### 3.6. Distribution of the Adoption Rate in Relation to the Biosecurity Components and Administrative Location of Fish Farms

The distribution of the adoption rate in relation to the biosecurity components and administrative location of fish farms is presented in Table 5. The overall adoption rate was intermediate (40.40 ± 30.10%) and ranged from 0 to 96.97%. Irrespective of the farm location, the isolation component (60.17 ± 25.68%) was significantly (*F* = 3.78; *p*=0.040) the most adopted followed by the traffic control (53.53 ± 34.86%) and sanitation (29.84 ± 26.00%). Whatever the location of the farm, the adoption rate of the biosecurity components was intermediate and showed an insignificant (*F* = 0.17; *p*=0.953) variation from 36.11 ± 39.22% (Douala I) to 43.05 ± 38.67% (Douala II). In addition, the adoption rates of the biosecurity components were not significantly (*p* > 0.05) different in the Douala III and IV subdivisions.

### 3.7. Adoption Rate as per the Biosecurity Measures

The adoption rate as per the biosecurity measures as highlighted in Table 6 shows that the less adopted (0%) measures were veterinary intervention, incineration of dead fish, and captured fish put back into water while breeding infrastructures are layout in derivation was the most adopted (96.7%).

### 3.8. Distribution of the Compliance Rate in Relation to the Socioeconomic and Demographic Characteristics of Fish Farmers

The distribution of the compliance rate in relation to the socioeconomic and demographic characteristics of fish farmers is summarized in Table 7. Of the characteristics studied, only age of fish farmers, level of education, and training received in fish farming significantly affected the compliance rate. Indeed, more biosecurity measures were observed by fish farmers whose age ranged from 18 to 40 years (45.24 ± 14.75%), who attended higher school (43.83 ± 14.44%), and who received training in fish farming (47.44 ± 14.39%).

### 3.9. Effects of the Compliance Rate on the Zootechnical Characteristics of the Farms

The effect of the compliance rate on the zootechnical characteristics of the farms is summarized in Table 8. An insignificant (*p* > 0.05) increase of the mortality rate and the productivity of farms with the compliance rate of biosecurity measures were noticed. In addition, the increase of the compliance rate negatively affected (*p* > 0.05) the health status of fish, while the low compliance rate rather positively influenced (*p*=0.01) the fish health. No significant correlation (*r* = −0.00; *p*=0.984) was found between the productivity of fish farms and the cost of the biosecurity practice.

### 3.10. Relationship between Fish Farm Characteristics and Implementation of Biosecurity Measures

Multivariate linear regression analysis of factors affecting the implementation of biosecurity measures in the fish farms of the Wouri division (Table 9) shows a strong (*R*^2^ = 0.725), positive, and significant (*p*=0.019) relationship between the level of education and the compliance rate. The health status of fish was weakly (*R*^2^ = 0.207), positively, and significantly (*p*=0.017) influenced by the compliance rate of biosecurity measures. A positive and nonsignificant (*p* > 0.05) relationship was observed with the sex of fish farmers, their religion, main occupation, cost of the biosecurity practice, training place, and the mortality rate. The marital status, farm age, purpose of fish farming, constraints related to the biosecurity practice, land acquisition method, training in fish farming, and the farm productivity were strongly, negatively, and nonsignificantly associated with the compliance rate of the biosecurity practice.

### 3.11. Biosecurity Affinities and Interactions between Fish Farms of the Subdivisions of the Wouri Division

**Figure 4:**
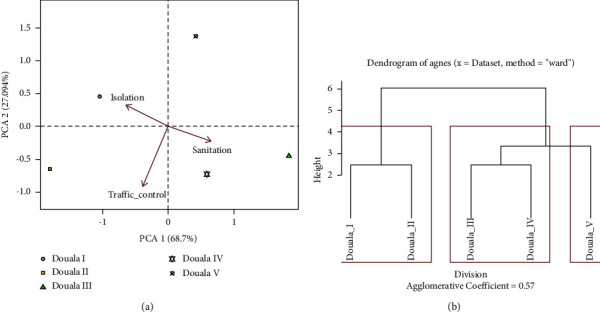
(a) Illustration of the principal component analysis of biosecurity affinities between farms of the subdivisions of the Wouri division. (b) The cluster plot showing the biosecurity practice similarities between fish farms of the subdivisions of the Wouri division.

The biosecurity affinities and interactions between farms of the subdivisions of the Wouri division (Figures 4(a) and 4(b)) show 95.79% of the variance in the data that was explained by the two principal component analysis (PCA) axes. The factorial axis PCA_1_ expressing 68.7% of the total variance revealed three groups of relationships between the subdivisions and the biosecurity components after projection. The first group including farms of Douala I and II subdivisions showed an affinity for the biosecurity components related to isolation and traffic control. The second group of similarity included farms of Douala III and IV having an affinity for the sanitation component while fish farms of Douala V belonging to the third group did not provide any relevant information on the biosecurity practice.

## 4. Discussion

The results of the socioeconomic and zootechnical characteristics of fish farming and biosecurity practices in the administrative division of Wouri, Cameroon showed that more than half of the farmers (63.64%) were between 18 and 40 years old, suggesting that this age group is young and therefore more active and productive. People over 40 years of age were less involved in fish farming probably because either that activity was secondary or these people were less dynamic. This result is comparable to 28.8% of fish farmers over the age of 40 observed by Olasunkanmi [20] in Osun State in Nigeria. The high involvement of the youth in fish farming is contradictory to the reports of Ngueguim et al. [9], Hirigoyen et al. [21], Adebayo Ot et al. [22], and Tiogué et al. [23] who highlighted that more than 40% of fish farmers were over 50 years old. The high representation of men (93.94%) in fish farming is in accordance with the findings of Ngueguim et al. [9], Tiogué et al. [23], and Bouelet Ntsama et al. [24] would be related to the socioeconomic constraints faced by women such as difficulties of access to land, lack of capital, poor management skills, and lack of credit opportunities. The proportion of trained fish farmers (39.39%) was higher than 5 and 8% reported, respectively, by Tiogué et al. [23] and Hirigoyen et al. [21]. The reason would be the geographical proximity of the Wouri division with the Institute of Fisheries and Aquatic Sciences (ISH) of the University of Douala at Yabassi whose purpose is to train fisheries engineers. For instance, out of 39.39% of trained farmers, 53.85% came from that higher school.

Although the high cost of the biosecurity practice (57.57% of farmers) was the main obstacle, there is no guarantee that biosecurity measures would be applied even if the cost was low given that farmers do not understand the importance and relevance of the biosecurity practice. The high percentage (42.42%) of farms in which fish showed clinical signs of diseases and the low proportion of farms (12.12%) with high productivity (>300 kg/year/m^3^) would be due to the lack of the biosecurity practice, poor farm management, and lack of finance. Though high, the proportion of farms showing clinical signs of fish infection was below the expected value and likely to increase because of the lack of training in fish disease diagnosis and the inattention of most farmers claiming not to observe abnormalities in fish. The high mortality rate (>15%) of fish recorded by 66% of the audited farms is probably attributed to the lack of training in fish farming resulting in the nonobservance of biosecurity measures at the origin of fish diseases. The untrained farmers can receive training on biosecurity practices at the Institute of Fisheries and Aquatic Sciences of the University of Douala, Cameroon.

The overall compliance rate (40.52 ± 14.70%) was intermediate and below the expected high level. In other words, the farms of the Wouri division were of type B or at a moderate risk level of contamination by pathogenic agents, hence the appearance of clinical signs of disease and the high rates of fish mortality observed in this study. This result would be due to financial constraints, ignorance, and negligence observed, respectively, in 57.57%, 15.15%, and 9.09% of fish farmers. The overall compliance rate is comparable to that reported by Kone et al. [25] and different from the low values noted by Obosi and Agbeja [7], Ngueguim et al. [9], Kouam and Moussala [18], Boutin [26], and Ricou [27]. The reason for this difference would be the increase in the proportion of fish farmers who have received training and the difference between sociodemographic and technical-economic conditions. The isolation component was highly and significantly the most observed followed by traffic management and sanitation because the measures relating to isolation seem inexpensive and less restrictive. This trend is contrary to that outlined by Ngueguim et al. [9], Kouam et al. [19], and Kone et al. [25] as which the most observed component was traffic management followed by isolation and sanitation and which was justified by the fact that traffic management has few measures compared to the other biosecurity components.

With respect to the frequency distribution of farms according to the compliance rate of biosecurity components, no farm (0%) was at a minor contamination risk level or recorded a high compliance rate (75–100%) of biosecurity components. This could be explained by the fact that producers face financial constraints, hence the absence of rigorous observance of biosecurity measures. Indeed, this study showed that the financing of the biosecurity practice was constraining for 57.57% of fish farmers. In Cameroon, there is no report on the good biosecurity practice in fish farms. However, Kouam et al. [19] reported that 9.74% of pig farms had the good biosecurity practice in the West Region of Cameroon and further because that minority of farmers (9.74%) would not have financial constraints or would be well trained in pig farming.

The overall adoption rate was intermediate (40.40 ± 30.10%) and varied from 0 to 96.97% due to financial constraints and lack of knowledge or training in biosecurity. Indeed, up to 60.61% of fish farmers in the Wouri division have not been trained in fish farming. The most adopted component was isolation followed by traffic control and sanitation because the isolation component would be technically and financially less constraining [9, 18, 19, 25]. On the other hand, for same reasons, the latter authors highlighted that traffic control was the most adopted followed by sanitation and isolation. The least adopted (0%) biosecurity measure were “veterinary visit,” “dead fish incinerated,” and “fish caught not returned to the water,” while the most adopted (96.97%) was “breeding infrastructures in derivation.” Certain measures are unknown or neglected by fish farmers or the latter lack finance to adopt other measures.

The analysis of the multivariate linear regression of the factors affecting the implementation of biosecurity showed positive and significant relationship between the level of education, the health status of the fish, and the compliance rate of biosecurity measures. A strong, positive, and significant relationship between the education level, the herd size, and the level of biosecurity adoption has been reported in beef cattle farms [28]. Ideally, the compliance rate would have negatively affected the health status of the fish. Hygiene measures would therefore not have the same effectiveness against infectious agents; the most effective would be the most restrictive and the least applied by fish farmers. The affinities of the biosecurity practice observed between the farms of certain subdivisions of the Wouri division would reflect a sociodemographic and technical-economic rapprochement.

The issue of compliance with hygiene measures on farms is undeniable because a good practice will allow the ISO certification of farms and will therefore ensure the sanitary quality of fish and customers. This study will certainly help stakeholders in the aquaculture sector in Cameroon and other countries with comparable farming systems to improve the level of the biosecurity risk of farms. This will reduce epizootics and optimize production.

## 5. Conclusion

The present study on the characteristics of fish farming and biosecurity practices in the division of Wouri, Cameroon, revealed that fish farming is an income-generating activity that still requires socioeconomic, technical, and institutional efforts for optimal productivity. Overall, the compliance and adoption rates of biosecurity measures were intermediate. No type C farms were recorded. The compliance rate was significantly affected by the age of fish farmers, the level of education, and training received in fish farming. The biosecurity measures to be improved were “veterinary visit,” “dead fish incinerated,” and fish caught not returned to water.” A significant positive relationship was established between the education level, fish health status, and biosecurity compliance rate. Biosecurity affinities have been observed between certain farms. The government should take the issue of aquaculture biosecurity very seriously by emphasizing on the education, training, and capacity building of farmers on biosecurity practices.

## Figures and Tables

**Table 1:** Typology of fish farms according to the compliance rate of biosecurity measures.

CR	Implementation level	Biosecurity practice/status	Risk ranking	Type of farms
(0–25)	Low	Poor	Major	A
]25−75]	Intermediate	Intermediate	Moderate	B
]75–100]	High	Good	Minor	C

CR: compliance rate.

**Table 2:** Distribution of farm frequencies according to the socioeconomic and demographic characteristics of farmers in the Woori division.

Socioeconomic and demographic characteristics	Modalities (*N* = 33)	Frequencies (%)
Farmer's age (years)	(18–40)	63.64
	>40	36.36

Sex	Male	93.94
	Female	6.06

Marital status	Married	54.54
	Single	42.42
	Widow(er)	3.03
	Divorced	0.00

Farm age (years)	(0–5)	87.89
	]5–10]	9.09
	>10	3.03

Level of education	Never been to school	0.00
	Primary	0.00
	Secondary	24.24
	Higher	75.76

Training in fish farming	Yes	39.39
	No	60.61

Training place on fish farming	ISH^1^	53.85
	Elsewhere	46.15

Main occupation	Fish farmer	39.39
	Crop and livestock producer	6.06
	Others	54.54

Purpose of fish farming	Autoconsumption	0.00
	Income generation	96.97
	Autoconsumption + income generation	3.03

Constraints related to biosecurity practice	Expensiveness	57.57
	Negligence	9.09
	Ignorance	15.15
	Expensiveness + negligence + ignorance	18.18

Cost of biosecurity practice (USD/production cycle)	(0–50)	33.33
	>50	18.18
	Unknown	48.48

Land acquisition method	Purchase	57.57
	Inheritance	27.27
	Renting	12.12
	Donation	3.03

N: number of farms; ^1^: Institute of Fisheries and Aquatic Sciences, University of Douala, Cameroon; USD: United States dollars.

**Table 3:** Distribution of farm frequencies according to the zootechnical characteristics in the Wouri division.

Zootechnical characteristics	Modalities (*N* = 33)	Frequencies (%)
Clinical signs of fish infection^1^	Yes	42.42
	No	57.58

Mortality rate of fish (%/production cycle)	]0–15]	34
	]15–30]	44
	]30–70]	22

Productivity (Kg/year/m^3^)	(0–150)	63.64
	]150–300]	24.24
	>300	12.12

^1^: ethological, anatomical, and physiological diagnosis based on skills acquired by farmers during the fish training program attended; N: number of farms.

**Table 4:** Distribution of the compliance rate per subdivisions of the Wouri division, Cameroon.

Subdivisions	*n*	Minimum	Maximum	Mean ± SD	*F*	*p*
Douala I	3	25.00	54.17	36.11 ± 15.78	0.167	0.953
Douala II	3	29.16	66.67	41.66 ± 21.66		
Douala III	11	16.67	75.00	42.80 ± 18.08		
Douala IV	9	20.83	58.33	40.74 ± 12.81		
Douala V	7	16.67	50.00	38.09 ± 11.13		

Total	33	16.67	75.00	40.52 ± 14.71	—	—

*n*: number of fish farms; SD: standard deviation.

**Table 5:** Distribution of the adoption rate in relation to the biosecurity components and administrative location of fish farms.

Biosecurity components	*Location of the farms (subdivisions)*					Overall (*N* = 33)	*F*	*p*
	Douala I (*n* = 3)	Douala II (*n* = 3)	Douala III (*n* = 11)	Douala IV (*n* = 9)	Douala V (*n* = 7)			
Isolation	66.67 ± 47.14 (0–100)	76.19 ± 37.09 (0–100)	54.54 ± 25.71 (18.18–100)	57.14 ± 36.53 (11.11–100)	65.30 ± 25.89 (28.57–100)	60.17 ± 25.68 (15.15–96.97)	0.41	0.800

Traffic control	55.56 ± 50.92 (0–100)	77.78 ± 38.49 (33.33–100)	51.51 ± 36.74 (9.09–72.72)	62.96 ± 32.08 (44.44–100)	33.33 ± 45.92 (0–85.71)	53.53 ± 34.86 (18.18–87.88)	0.46	0.762

Sanitation	16.67 ± 17.29 (0–33.33)	19.05 ± 17.12 (0–33.33)	35.05 ± 30.01 (0–81.81)	26.98 ± 31.34(0–77.78)	25.51 ± 29.21 (0–85.71)	29.84 ± 26.00 (0–72.73)	1.10	0.363

Overall	36.11 ± 39.22 (0–100)	43.05 ± 38.67 (0–100)	42.80 ± 29.79 (0–100)	40.28 ± 35.41 (0–100)	38.09 ± 34.14 (0–100)	40.40 ± 30.10 (0–96.97)	0.17	0.953

*F*	6.08	13.54	1.16	2.76	4.06	3.78	—	—
*p*	**0.01** ^*∗*^	**0.001** ^*∗*^	0.332	0.086	**0.032** ^*∗*^	**0.040** ^*∗*^	—	—

Mean ± standard deviation (minimum-maximum); *n*: number of audited fish farms in the subdivision; *N*: total number of audited farms in the Wouri division; ^*∗*^: significant. Bold values are used to indicate the significant probability of the error i.e., when *p* is significant.

**Table 6:** Adoption rate as per the biosecurity measures.

No	Biosecurity component in relation to isolation	*n* (the adoption rate in %)
1	Farm is fenced	26 (78.79)
2	Other animals species are absent on the farm	18 (54.54)
3	New fish are quarantined before rearing	18 (54.54)
4	Absence of bushes and trees around farms	17 (51.51)
5	Space for visitors	5 (15.15)
6	Water flow is continuous	23 (69.70)
7	Culture facilities are layout in derivation	32 (96.97)

*Biosecurity component in relation to traffic control*		
8	Visitors not allowed to have contact with water	18 (54.54)
9	No exchange of breeding tools between farms	29 (87.88)
10	Water supply tracks protected to trap debris and unwanted aquatic animals	6 (18.18)

*Biosecurity component in relation to sanitation*		
11	Use of footbaths	5 (15.15)
12	Veterinary intervention	0 (0.00)
13	Incineration of dead fish	0 (0.00)
14	Especial outfit (clean coverall and boots) for staff	3 (9.09)
15	Especial outfit for visitors	1 (3.03)
16	Analysis of water quality	1 (3.03)
17	Diagnosis of fish diseases^1^	14 (42.42)
18	Sanitary lock	21 (63.64)
19	Awareness of biosecurity measures	18 (54.54)
20	Awareness of fish diseases	11 (33.33)
21	Disinfection of breeding tools before use	15 (45.45)
22	Disinfection of breeding tools after use	24 (72.73)
23	Treatment of fish diseases	15 (45.45)
24	Captured fish put back into water	0 (0.00)

^1^: ethological, anatomical, and physiological diagnosis based on skills acquired by farmers during fish training program attended; *n*: number of fish farms.

**Table 7:** Distribution of the compliance rate in relation to the socioeconomic and demographic characteristics of fish farmers in the Wouri division, Cameroon.

Characteristics	Modalities	*Compliance rate (mean* *±* *standard deviation) of biosecurity components*				*U*	*p*
		Isolation	Traffic control	Sanitation	Total		
Age groups (years)	[18–40]	60.54 ± 22.09	50.79 ± 29.10	36.05 ± 17.85	45.24 ± 14.75	60.50	**0.015** ^*∗*^
	>40	59.52 ± 15.92	58.33 ± 20.72	13.09 ± 13.56	32.29 ± 10.83		

Sex	Male	60.83 ± 19.51	52.69 ± 25.50	28.11 ± 19.94	40.86 ± 14.33	—	—
	Female	50.00 ± 30.31	66.67 ± 47.14	21.43 ± 20.20	35.42 ± 26.52		

Marital status	Married	64.29 ± 15.69	55.56 ± 25.57	19.44 ± 16.94	37.04 ± 12.93	77.50	0.067
	Single	57.14 ± 23.10	52.38 ± 28.39	39.79 ± 17.20	46.73 ± 14.54		
	Widow (er)	28.57 ± 0	33.33 ± 0	7.14 ± 0	16.67 ± 0	—	—

Level of education	Secondary	50.00 ± 15.27	41.67 ± 29.55	17.86 ± 17.50	30.21 ± 10.21	45.50	**0.022** ^*∗*^
	Higher	63.43 ± 20.24	57.33 ± 24.57	30.85 ± 19.64	43.83 ± 14.44		

Training received in fish farming	Yes	57.14 ± 25.42	53.85 ± 28.99	41.21 ± 14.91	47.44 ± 14.39	69.00	**0.025** ^*∗*^
	No	62.14 ± 15.56	53.33 ± 25.13	18.93 ± 17.54	36.04 ± 13.40		

Training place in fish farming	ISH^1^	54.76 ± 27.73	66.67 ± 29.82	42.86 ± 9.04	49.31 ± 13.55	4.00	0.243
	Elsewhere	47.62 ± 8.25	55.56 ± 19.25	23.81 ± 14.87	34.72 ± 12.03		

Longevity of the farm (years)	(0–5)	60.59 ± 19.71	54.02 ± 25.84	29.06 ± 20.47	41.52 ± 14.92	41.50	0.038
	>5	57.14 ± 23.33	50.00 ± 33.34	17.86 ± 9.22	33.33 ± 12.26		

Main occupation	Fish farmer	54.94 ± 20.90	56.41 ± 34.39	36.26 ± 16.36	44.23 ± 11.10	*F* = 1.78	0.189
	Crop and livestock producer	57.14 ± 40.40	33.33 ± 0	10.71 ± 5.05	27.09 ± 14.73		
	Others	62.24 ± 18.25	52.38 ± 17.12	19.90 ± 21.18	36.61 ± 16.52		

Land acquisition method	Purchase	63.16 ± 19.23	49.12 ± 25.75	24.06 ± 19.23	38.60 ± 15.52	*F* = 1.03	0.369
	Inheritance	61.90 ± 20.20	70.37 ± 26.06	33.33 ± 20.82	46.76 ± 14.55		
	Renting	50.00 ± 18.44	41.67 ± 16.67	30.36 ± 23.60	37.50 ± 11.29		
	Donation	28.57 ± 0	33.33 ± 0	35.71 ± 0	33.00 ± 0	—	—

Constraints related to biosecurity practice	Expensiveness^2^	59.40 ± 20.90	56.14 ± 29.51	37.22 ± 17.43	46.27 ± 15.14	*F* = 2.83	0.055
	Negligence^3^	52.38 ± 29.74	44.44 ± 19.25	28.57 ± 12.37	37.50 ± 4.17		
	Ignorance^4^	54.29 ± 18.63	53.33 ± 29.82	14.28 ± 13.36	30.83 ± 12.71		
	2 + 3 + 4	71.43 ± 9.04	50.00 ± 18.26	8.33 ± 14.58	31.94 ± 10.43		

Cost of biosecurity practice (USD/production cycle)	[(0–50)	64.93 ± 24.21	45.45 ± 22.48	34.41 ± 20.48	44.70 ± 15.38	*F* = 2.31	0.117
	>50	54.76 ± 21.03	66.67 ± 36.51	39.28 ± 16.13	47.22 ± 11.98		
	Unknown	58.93 ± 16.39	54.17 ± 23.96	18.75 ± 16.68	35.16 ± 13.94		

^1^: Institute of Fisheries and Aquatic Sciences, University of Douala, Cameroon; *p*: significance level; ^*∗*^: significant; *U*: Mann–Whitney test value; *F*: analysis of variance value; USD: United; States dollars. Bold values are used to indicate the significant probability of the error i.e., when *p* is significant.

**Table 8:** Effects of the compliance rate on the zootechnical characteristics of the farms.

Compliance rate (%)	*Zootechnical characteristics of fish farms*				
	Mortality rate (% of dead fish/production cycle)	Productivity (Kg/year/m^3^)	*Fish health status*		
			+	−	*p*
[0–15]	16.83 ± 10.01 (1–30)	110.80 ± 80.19 (0.096–200)	0%	100%	**0.01** ^*∗*^
[15–70]	18.94 ± 13.32 (2–60)	163.10 ± 175.10 (2–571)	51.85%	48.15%	1.00

*U*	79.50	75.00	No value of Fisher exact's test		
*p*	0.963	0.797	0.158	0.318	

−: % of farms with no clinical signs of infection of fish; +: % of farms with clinical signs of infection of fish; ^*∗*^: significant. Bold values are used to indicate the significant probability of the error i.e., when *p* is significant.

**Table 9:** Results of the multivariate linear regression analysis between the socioeconomic characteristics of fish farmers and the zoo technical characteristics of farms in the Wouri division, and the compliance rate of biosecurity measures.

Characteristics	Regression coefficients	*p*	*R* ^2^	Constant
*Socioeconomic and demographic characteristics*				
Farmer's age	−11.745	0.367	MR^2^ = 0.725 AR^2^ = 0.169	28.937
Sex	2.550	0.886		
Marital status	−14.035	0.467		
Farm age	−14.567	0.378		
Level of education	29.022	**0.019** ^*∗*^		
Main occupation	12.295	0.870		
Purpose of fish farming	−24.216	0.101		
Constraints related to biosecurity practice	−1.279	0.849		
Cost of biosecurity practice	18.007	0.417		
Land acquisition method	−20.830	0.469		
Training in fish farming	−11.535	0.329		
Training place in fish farming	9.596	0.286		

*Zootechnical characteristics*				
Fish health status^1^	13.277	**0.017** ^*∗*^	MR^2^ = 0.207 AR^2^ = 0.125	36.134
Mortality rate	0.026	0.898		
Productivity	−11.232	0.470		

MR^2^: multiple *R*-squared; AR^2^: adjusted R-squared; R^2^: determination coefficient; *p*: error probability; ^*∗*^: significant (*p* < 0.05); ^1^: onset of clinical signs of disease.

## Data Availability

The data used to support the findings of this study are available upon request to the corresponding author.
